# Effects of linoleic acid and its trans-10 biohydrogenation intermediates (trans-10, cis-12 conjugated linoleic acid and trans-10 18:1) on lipogenic gene expression and Δ9-desaturation indices in bovine intramuscular adipocytes

**DOI:** 10.1093/jas/skag268

**Published:** 2026-08-26

**Authors:** Maykal N Tsonov, Perri E Gish, Payam Vahmani

**Affiliations:** Department of Animal Science, University of California, Davis, Davis, CA 95616, United States; Department of Animal Science, University of California, Davis, Davis, CA 95616, United States; Department of Animal Science, University of California, Davis, Davis, CA 95616, United States

**Keywords:** beef, trans-10 shift, marbling, Δ9-desaturation

## Abstract

Trans-10, cis-12 conjugated linoleic acid (t10, c12-CLA) and trans-10 18:1 (t10-18:1) are major ruminal biohydrogenation intermediates of linoleic acid (LNA) under high-grain diets. T10, c12-CLA has been shown to reduce Δ9-desaturase (SCD1) activity and the cis-monounsaturated to saturated fatty acid ratio (cisMUFA/SFA) in beef fat. However, the bioactivities of LNA and t10-18:1 remain poorly understood. This study evaluated LNA and its trans-10 intermediates in bovine intramuscular adipocytes. Intramuscular preadipocytes were isolated from three feedlot-finished Angus steers and differentiated for two experiments. Experiment 1 evaluated dose-response effects of t10-18:1, t10, c12-CLA, and LNA at 0, 25, 50, and 100 μM. Experiment 2 compared LNA, t10-18:1, t10, c12-CLA, and c10-18:1 at 50 μM with the bovine serum albumin (BSA) control. Lipid accumulation, gene expression, and fatty acid composition were analyzed by Nile Red staining, qPCR, and gas chromatography, respectively. Data were analyzed using the MIXED procedure of SAS. Lipid accumulation was unaffected by treatment in either experiment. T10-18:1 dose-dependently increased SCD1 expression and c9-18:1/18:0 in Experiment 1 (*P* < 0.05), with both greater than the BSA control and other treatments in Experiment 2 (*P* < 0.05). LNA dose-dependently reduced SCD1 expression, cisMUFA/SFA, c9-16:1/16:0, and c9-18:1/18:0 in Experiment 1, with all four lower than the BSA control in Experiment 2 (*P* < 0.05). T10, c12-CLA reduced SCD1 expression in both experiments and cisMUFA/SFA in Experiment 1 (*P* < 0.05) but did not alter c9-16:1/16:0 or c9-18:1/18:0 in either experiment. These findings demonstrate distinct effects of LNA and its trans-10 biohydrogenation intermediates on lipogenic gene expression and Δ9-desaturation in bovine intramuscular adipocytes.

## Teaser

Linoleic acid and its rumen-derived metabolites can differently regulate fat metabolism and fatty acid composition in bovine intramuscular fat cells.

## Graphical Abstract

**Graphical Abstract:**
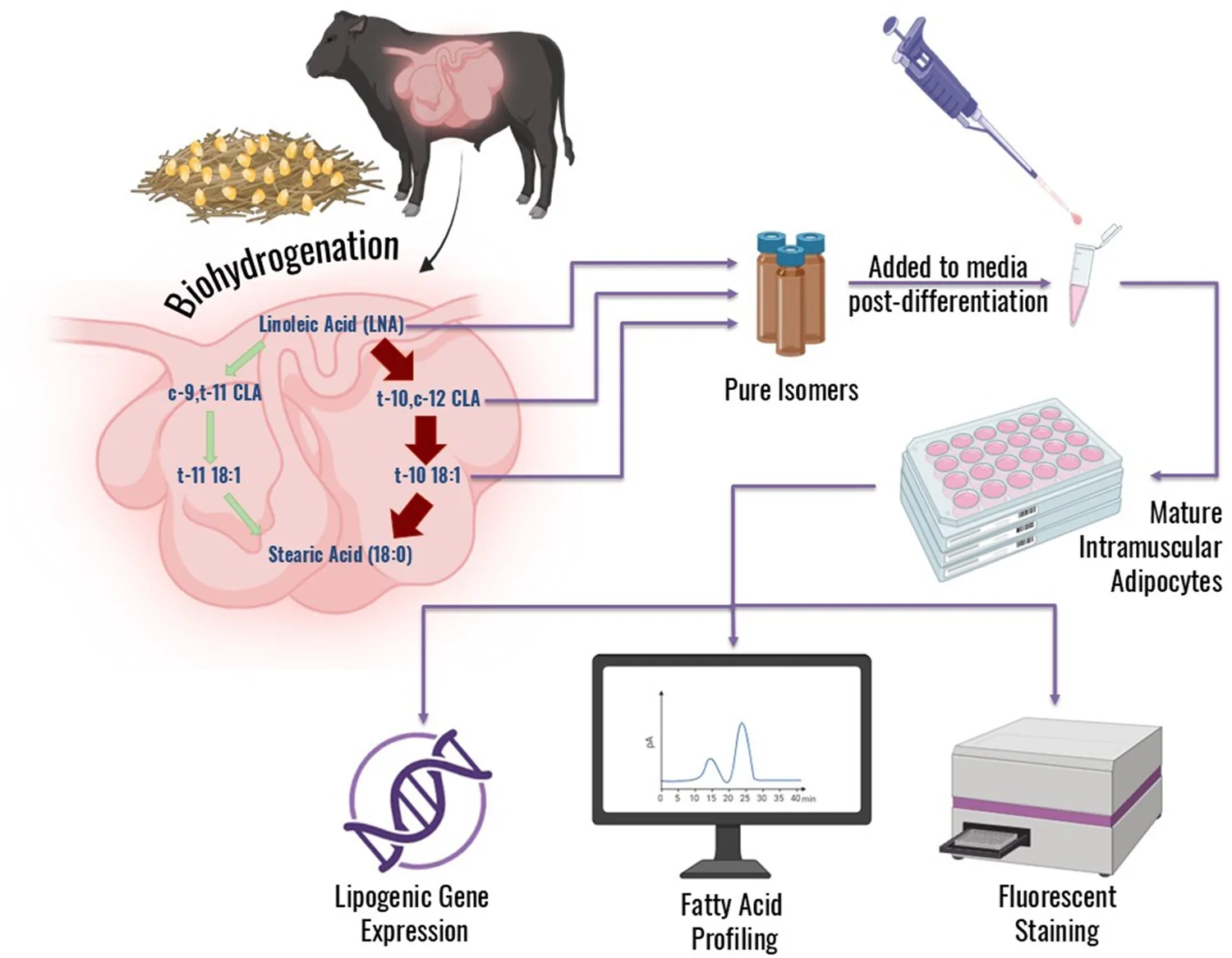

## Introduction

In feedlot production systems, high-grain diets are commonly fed for extended periods to promote growth performance, improve feed efficiency, and enhance carcass characteristics, including marbling and overall meat quality (Park et al. 2018). High-grain feeding has also been associated with an increased cis-monounsaturated fatty acid (cisMUFA) to saturated fatty acid (SFA) ratio in beef fat, an important indicator of fat quality related to palatability and healthfulness (Smith et al. 2009, 2020; Gotoh and Joo 2016; Ladeira et al. 2018; Smith 2024). This response is primarily attributed to increased expression and activity of stearoyl-CoA desaturase-1 (SCD1), which catalyzes the Δ9 desaturation of SFA to cisMUFA, particularly the conversion of stearic acid (18:0) to oleic acid (cis-9 18:1) (Smith et al. 2009; Gotoh and Joo 2016; Ladeira et al. 2018).

Corn grain is the primary energy source in U.S. feedlot rations, comprising 60% to 85% of dietary dry matter in finishing diets. Corn lipids are a major source of linoleic acid (cis-9, cis-12 18:2; LNA), which accounts for approximately 55% to 60% of total fatty acids in corn-based diets (Vahmani et al. 2021). Within the rumen, dietary unsaturated fatty acids, including LNA, undergo extensive microbial biohydrogenation, resulting in the formation of saturated end-products, primarily stearic acid, as well as a range of biohydrogenation intermediates, including conjugated linoleic acid (CLA) and trans 18:1 isomers (Alves et al. 2021). A proportion of these intermediates escapes complete biohydrogenation and is incorporated into tissue lipids following post-ruminal absorption. Under forage-based conditions, LNA is predominantly biohydrogenated into cis-9, trans-11 CLA (c9, t11-CLA) and trans-11 18:1 (vaccenic acid; t11-18:1), which are subsequently reduced to stearic acid. In contrast, high-grain feedlot diets can alter ruminal microbial populations, resulting in a shift in biohydrogenation pathways toward the trans-10 pathway (the “trans-10 shift”), characterized by increased formation of trans-10, cis-12 CLA (t10, c12-CLA) and trans-10 18:1 (t10-18:1) (Vahmani et al. 2025).

In dairy cows, dietary conditions that promote a trans-10 shift (e.g., diets high in starch or unsaturated fatty acids) are associated with milk fat depression and consequent economic losses due to reduced milk fat yield (Vahmani et al. 2025). This diet-induced milk fat depression is largely attributed to the antilipogenic actions of t10, c12-CLA through downregulation of lipogenic gene expression in the mammary gland. Beyond its role in milk fat depression, t10, c12-CLA is known for its potent anti-adipogenic and antilipogenic effects in both in vivo rodent models and in vitro adipocyte cell culture studies (Lehnen et al. 2015). In primary bovine adipocyte cultures, t10, c12-CLA has been shown to suppress adipocyte differentiation and downregulate lipogenic enzymes, including fatty acid synthase (FAS) and SCD1, resulting in reduced de novo fatty acid synthesis and a lower cisMUFA/SFA ratio (Chung et al. 2006, Kadegowda et al. 2013).

Because of its antilipogenic effects, it has been suggested that dietary conditions leading to excess ruminal production of t10, c12-CLA may negatively affect marbling deposition in feedlot cattle (Smith et al. 2006). However, in vivo studies using rumen-protected t10, c12-CLA or feeding conditions that promote a trans-10 shift (e.g., high-grain diets supplemented with plant oils) have shown no negative effects on marbling score but do consistently reduce SCD1 expression and the cisMUFA/SFA ratio in beef fat (Joseph et al. 2010; Schlegel et al. 2012; Oliveira et al. 2014; Choi et al. 2016; Teixeira et al. 2017). Moreover, Vahmani et al. (2025) recently reported that both t10, c12-CLA and t10-18:1 are negatively correlated with the cisMUFA/SFA ratio in intramuscular fat from feedlot cattle. Notably, t10-18:1 is the predominant trans fatty acid in blood and tissues of grain-fed cattle, often present at concentrations 10-20-fold higher than t10, c12-CLA (Alves et al. 2021; Vahmani et al. 2025). However, the causal effects of t10-18:1 on lipogenesis in bovine adipocytes, as well as the role of LNA, the predominant polyunsaturated fatty acid (PUFA) in beef adipose tissues, remain uncharacterized.

To address this knowledge gap, the objective of the present study was to evaluate the effects of LNA and its trans-10 biohydrogenation intermediates (t10-18:1 and t10, c12-CLA) on lipid accumulation, expression of genes involved in lipid metabolism, and fatty acid composition in differentiated bovine intramuscular adipocytes. We hypothesized that t10-18:1 exerts antilipogenic effects comparable to t10, c12-CLA in cultured adipocytes, whereas LNA does not alter lipid accumulation or the expression of lipogenic genes. To test this hypothesis, two independent experiments were conducted. In Experiment 1, differentiated adipocytes were treated with different concentrations (0, 25, 50, and 100 μM) of LNA, t10-18:1, or t10, c12-CLA to characterize the dose-dependent effects of each fatty acid. In Experiment 2, differentiated adipocytes were treated for 9 d with a single concentration (50 μM) of LNA, t10-18:1, or t10, c12-CLA to directly compare their effects. A fourth treatment, c10-18:1, was included in Experiment 2 to determine whether the biological activity of t10-18:1 was attributable to double-bond geometry (trans vs. cis).

## Materials and methods

### Animals and cell culture

Three Angus steers (17–18 mo of age) were used in this study for cell isolations. Steers were finished at the UC Davis Feedlot, and tissues for cell isolations were collected post-slaughter under USDA inspection at the UC Davis Meat Lab. Approximately 100 g of brisket point (pectoralis superficialis) was removed within 30 min post-slaughter. The tissues were submerged in 70% ethanol, followed by three 5-min washes in 1× PBS, and then transported to the laboratory, where visible intramuscular fat was dissected under sterile conditions. Finely sliced intramuscular fat was used for cell isolation by collagenase digestion according to Yanting et al. (2018). The lysate was filtered through a 100 µm cell strainer (Corning Inc., Corning, NY, USA) and centrifuged at 500 × *g* for 5 min. The resulting pellet containing the stromal vascular fraction (SVF) was washed with DMEM (Cat. No. 11-965-092, Gibco) and seeded into 75 cm² culture flasks. The adherent SVF cells (hereafter referred to as preadipocytes) were cultured in high-glucose DMEM (Cat. No. 11-965-092, Gibco) supplemented with 10% fetal bovine serum (Cat. No. A5256801, Gibco), 2% antibiotic-antimycotic solution (Cat. No. 15240062, Gibco), and 0.5% gentamicin (Cat. No. 15710064, Gibco) in a water-jacketed incubator at 37 °C with 5% CO₂. The culture medium was replaced every 3 d. Following initial isolation, preadipocytes from each donor steer were divided into three subcultures and maintained separately through passage 4 before being used in both Experiments 1 and 2.

**Figure 1:**
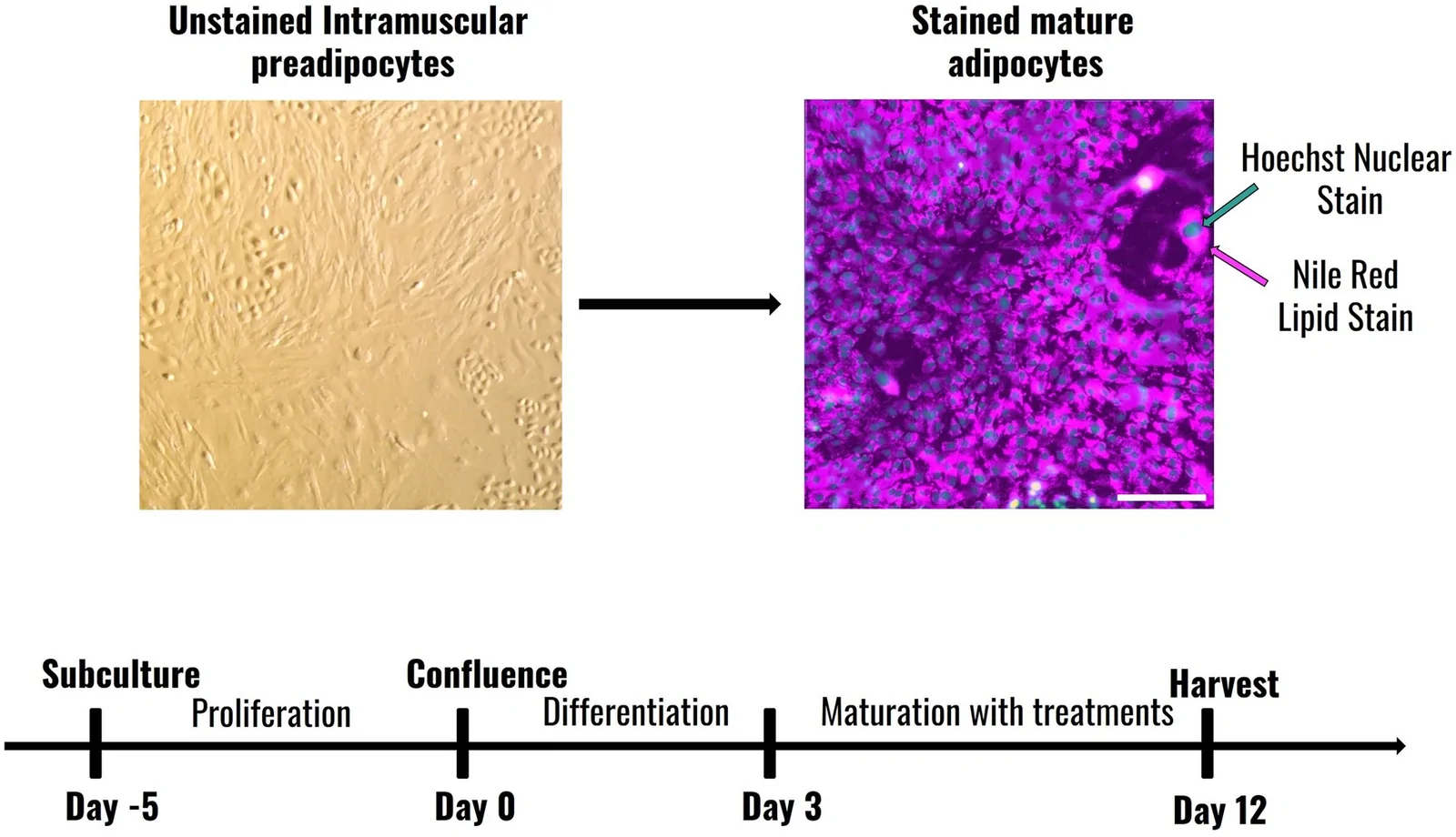
Timeline of intramuscular bovine adipocyte culture, differentiation, and treatments for Experiments 1 and 2. The adipocytes were differentiated for 3 d followed by 9 d in maturation media containing BSA-complexed fatty acids (treatments) prior to harvest prior to harvest. Neutral lipids and nuclei were visualized by Nile Red and Hoechst staining, respectively, on d 9 post-differentiation. Representative images were captured using a fluorescence microscope at 40× magnification.

For both cell culture experiments, passage-4 preadipocyte subcultures derived from three donor steers were seeded into 24-well plates, with each subculture distributed among 3–4 wells, and allowed to reach 100% confluence. Adipogenic differentiation was then induced for 3 d using high-glucose DMEM supplemented with 5% FBS containing 0.25 µM 3-isobutyl-1-methylxanthine (IBMX) (Cat. No. PHZ1124, Thermo Fisher), 500 µM dexamethasone (Cat. No. 08168, Sigma Aldrich), 5 µM troglitazone (Cat. No. T2573-5MG, Sigma Aldrich), 5.25 µM insulin (Cat. No. SLCJ7615, Sigma Aldrich), and 10 mM sodium acetate (Cat. No. CLM-156-5, Cambridge Isotope Laboratories). This was followed by a 9-d maturation phase using differentiation medium lacking IBMX and dexamethasone (ie, maturation medium), as adapted from Kadegowda et al. (2013). The maturation medium was replaced every 3 d, and fresh fatty acid treatments were applied at each medium change throughout the 9-d maturation period (Figure 1).

### Fatty acid preparation and addition to cell culture media

Linoleic acid was purchased from Nu-Chek Prep, Inc. (Elysian, MN, USA). *Trans-*10, cis-12 CLA was purchased from Larodan (Solna, Sweden). *Trans*-10-18:1 was purchased from Life Lipids (San Diego, CA, USA). *Cis*-10-18:1 was purchased from Toronto Research Chemicals (Toronto, Canada). All fatty acids were >97% pure according to the suppliers, confirmed by GC analysis. Fatty acids were complexed to 1 mM fatty acid-free bovine serum albumin (BSA) at a 4:1 molar ratio (ie, 4 mM fatty acid), as described by Evans et al. (2001), and were subsequently diluted in maturation medium to attain the desired final concentration. Control cell cultures received a BSA-vehicle (v/v). Cells were exposed to fatty acid treatments for 9 d post-differentiation.

In Experiment 1, differentiated adipocytes were treated for 9 d with LNA, t10, c12-CLA or t10-18:1 at final concentrations of 0 µM (BSA vehicle control), 25, 50, or 100 µM. In Experiment 2, differentiated adipocytes were treated for 9 d with the BSA vehicle control, LNA, t10, c12-CLA, t10-18:1, or c10-18:1, with all fatty acid treatments applied at a final concentration of 50 µM. This concentration was selected because it represented the intermediate dose evaluated in Experiment 1 and allowed direct comparison among fatty acid treatments. The following Methods sections were identical for Experiments 1 and 2: staining and assessment of cellular lipid accumulation, fatty acid analysis, and quantitative PCR.

### Staining and assessment of cellular lipid accumulation

At the end of the fatty acid treatment period (d 9 post-differentiation), lipid content in adipocytes was assessed by Nile Red staining. Culture medium was removed, and cells were fixed with 10% neutral buffered formalin for 45 min at room temperature. Each well was incubated with Nile Red neutral lipid stain (Cat. No. N1142, Invitrogen) and Hoechst nuclear stain (Cat. No. H21486, Thermo Fisher) for 20 min in the dark, according to the manufacturer’s instructions. Excess stain was removed by washing with 1× PBS, and composite images were acquired using a Leica DFC9000 GTC camera and LAS X software with a dry 40× objective to visualize lipid accumulation. Fluorescence intensity for Nile Red and Hoechst was also quantified using a microplate reader (Biotek Synergy H1). For Nile Red, excitation/emission wavelengths of 552/636 nm were used to detect neutral lipids, while 350/461 nm were used for Hoechst to visualize nuclei (Figure 1). The Nile Red fluorescence signal (lipid index) was normalized to Hoechst fluorescence (nuclear index) to account for variation in cell number and expressed as a lipid/nuclei ratio (Jurek et al. 2020).

### Fatty acid analysis

The fatty acid composition of adipocytes was determined at d 9 post-differentiation using gas chromatography (GC). Briefly, cells washed twice with 1× PBS followed by removal from culture plates via cell scrapers (Tisch Scientific) and freeze-drying, and fatty acids were converted to methyl esters via base-acid methylation using sodium methoxide followed by methanolic HCl (Alves et al. 2013). Cis-10 17:1 methyl ester (Nu-Chek Prep Inc.) was added as an internal standard prior to derivatization. The resulting fatty acid methyl esters (FAME) were analyzed using a CP-Sil 88 column (100 m × 0.25 mm ID × 0.2 μm film thickness) on a TRACE 1310 gas chromatograph (Thermo Fisher Scientific) equipped with a flame ionization detector (FID). Hydrogen was used as the carrier gas (1 mL/min). For each analysis, 1 μL of the sample was injected using a 20:1 split ratio at an inlet temperature of 250 °C. The oven temperature program was as follows: 45 °C for 4 min, increased to 175 °C at 13 °C/min and held for 27 min, then increased to 215 °C at 4 °C/min and held for 35 min (Kramer et al. 2008). The FID was maintained at 250 °C with air, hydrogen, and nitrogen flow rates of 300, 30, and 29 mL/min, respectively. Individual peaks were identified using reference standards (GLC-463 and GLC-603, Nu-Chek Prep Inc.) and retention times reported in the literature (Vahmani et al. 2016b, 2017). FAME were quantified from chromatographic peak areas relative to the internal standard (cis-10 17:1), and individual fatty acid concentrations were expressed as μg/well (Kadegowda et al. 2013).

### RNA isolation, cDNA synthesis, and quantitative PCR

Total RNA was extracted from cells at d 9 post-differentiation using TRIzol reagent (Cat. No. 15596026, Invitrogen) in combination with the RNeasy Plus Mini Kit (Cat. No. 74106, QIAGEN). Briefly, chloroform was added to the TRIzol homogenate at a 1:5 (v/v) ratio of chloroform to TRIzol, followed by centrifugation at 12,000 × *g* for 15 min at 4 °C to separate the phases. The upper phase was transferred to a new tube and mixed with an equal volume of 70% ethanol prior to RNA purification using the RNeasy Plus Mini Kit according to the manufacturer’s instructions. RNA quantity and quality were determined using a NanoDrop ND-2000 spectrophotometer (Thermo Fisher Scientific Inc.). Complementary DNA (cDNA) was synthesized using the Maxima First Strand cDNA Synthesis Kit (Cat. No. K1682, Thermo Fisher Scientific) according to the manufacturer’s instructions, using equal amounts of total RNA for all samples. Quantitative PCR (qPCR) reactions were prepared using 2× Universal SYBR Green Fast qPCR Mix (Cat. No. RK21203, ABClonal) and gene-specific primer pairs (Table 1). Cycle threshold (Ct) values were determined using an Applied Biosystems MiniAmp Thermal Cycler (Thermo Fisher Scientific). Relative expression of target genes was calculated using 2^−ΔCt^, where ΔCt was defined as the Ct value of the target gene minus the mean Ct value of the two housekeeping genes (GAPDH and UXT) (Schmittgen and Livak 2008). The 2^−ΔCt^ values were used for statistical analysis and are presented as fold change relative to the BSA control.

**Table 1:** Gene-specific forward and reverse primer sequences used for qPCR.

Gene	Forward primer (5′–3′)	Reverse primer (3′–5′)	Accession number
**GAPDH**	GATGCTGGTGCTGAGTATGT	GCAGAAGGTGCAGAGATGAT	XM_010844969.1
**UXT**	CAGCTGGCCAAATACCTTCAA	GTGTCTGGGACCACTGTGTCAA	NM_001037471.2
**SREBP-1c**	CTGGAGAGGCTGGACTGAGG	GCTTTCCCAAGACTCAGCAC	XM_027522661.1
**FAS**	TCATCCCCCTGATGAAGAAG	AGATGGTGGCAGAGGAGCTA	XM_070773861.1
**ACC**	AGCTGAATTTTCGCAGCAAT	GGTTTTCTCCCCAGGAAAG	XM_070772629.1
**PPARγ**	ATCTGCTGCAAGCCTTGGA	TGGAGCAGCTTGGCAAAGA	XM_027523658.1
**GLUT4**	CTACTCAGGACTGACGTCGGGACT	ACCTAGCACTGGGCGATTAGG	NM_174604.1
**SCD-1**	TTATTCCGTTATGCCCTTGG	GTTAGTTGTGGAAGCCCTCA	XM_070780654.1
**FABP4**	CAAATTGGGCCAGGAATTTGA	TCTCATAAACTCTGGTGGCAGTGAC	XM_027561259.1

GAPDH, glyceraldehyde 3-phosphate dehydrogenase; UXT, ubiquitously expressed prefoldin-like chaperone.

### Statistical analysis

Data from both experiments were analyzed as a randomized complete block design using the MIXED procedure (PROC MIXED) of SAS (SAS Institute Inc., Cary, NC, USA). Each donor steer was considered a block to account for biological variation among animals. Three independent passage-4 subcultures from each donor steer served as biological replicates, with subculture included as a random effect in the model. Normality of residuals from the PROC MIXED model was assessed using the Anderson-Darling test, and all datasets met the assumption of normality. In Experiment 1 (dose-response study), concentration was included as the fixed effect for each fatty acid, and orthogonal polynomial contrasts were used to test for linear and quadratic dose–response effects. In Experiment 2 (fatty acid comparison), fatty acid treatment was included as the fixed effect. For both experiments, pairwise comparisons among least-squares means were performed using the PDIFF option of the LSMEANS statement with Tukey’s adjustment for multiple comparisons. Results are presented as least-squares means ± standard error of the mean (SEM). Differences were considered significant at *P *< 0.05, and trends were declared at 0.05 ≤ *P *< 0.10.

## Results

### Experiment 1: dose–response of linoleic acid and its trans-10 intermediates in differentiated adipocytes

In the first experiment, we evaluated the bioactivity of LNA, t10, c12-CLA, and trans-10 18:1 by exposing differentiated adipocytes to increasing concentrations (0–100 μM) of each fatty acid and assessing their effects on cellular lipid content, fatty acid composition, and lipogenic gene expression.

#### Cellular lipid content and fatty acid composition

**Figure 2:**
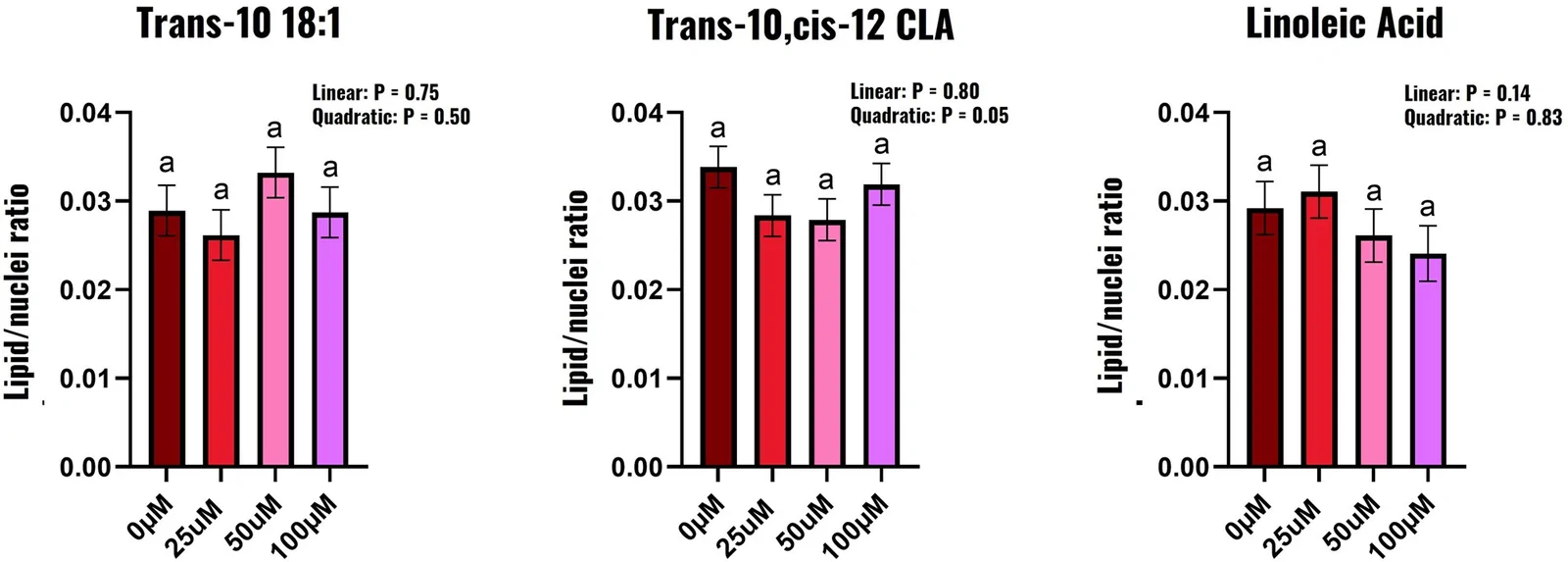
Normalized lipid accumulation (lipid/nuclei ratio) in mature intramuscular adipocytes after treatment with increasing doses (0–100 µM) of t10-18:1, t10, c12-CLA, and linoleic acid for 9 d. Linear and quadratic dose-response *P*-values are shown above each graph. Results are shown as mean ± SEM. Bars sharing a common letter are not significantly different (*P* > 0.05).

None of the fatty acid treatments affected normalized lipid content (lipid/nuclei ratio) in mature adipocytes, regardless of dose (Figure 2). Consistent with these findings, increasing doses of t10-18:1 (Table 2), LNA (Table 3), or t10, c12-CLA (Table 4) did not affect total fatty acid content (μg/well) in mature adipocytes. The effects of increasing doses of t10-18:1, LNA, and t10, c12-CLA on individual fatty acid amounts and major fatty acid classes are also presented in Tables 2–4. Increasing doses of t10-18:1 resulted in a linear increase in the amount of t10-18:1 (*P* < 0.01), with no changes in other individual fatty acids or major fatty acid classes (Table 2). Similarly, increasing doses of t10, c12-CLA resulted in a linear increase in the amount of t10, c12-CLA (*P* < 0.01), with no changes in other individual fatty acids or major fatty acid classes (Table 3). Increasing doses of LNA resulted in linear increases in the amount of LNA and total n-6 PUFA (both *P* ≤ 0.01), accompanied by linear decreases in total cis-MUFA (*P* = 0.03) and n-3 PUFA (*P* = 0.02), and a tendency for a reduction in c9-18:1 (*P* = 0.06; Table 4).

**Table 2:** Effects of increasing doses of trans-10 18:1 on fatty acid composition (μg/well) of mature bovine intramuscular adipocytes.^1^

Fatty acid	0 μM	25 μM	50 μM	100 μM	SEM	Linear *P*-value	Quadratic *P*-value
**16:0**	3.15	2.66	2.98	2.68	0.19	0.21	0.68
**18:0**	1.79	1.69	1.51	1.51	0.18	0.27	0.59
**c9-16:1**	0.09	0.05	0.07	0.09	0.02	0.73	0.17
**c9-18:1**	0.54	0.55	0.60	0.60	0.05	0.33	0.75
**c10-18:1**	n.d.	n.d.	n.d.	n.d.	N/A	N/A	N/A
**t10-18:1**	0.00^a^	0.82^ab^	1.90^bc^	2.02^c^	0.30	<0.01	0.03
**t10, c12-CLA**	n.d.	n.d.	n.d.	n.d.	N/A	N/A	N/A
**LNA**	0.18	0.20	0.22	0.21	0.02	0.24	0.27
**∑SFA**	6.11	5.91	5.37	5.17	0.76	0.36	0.83
**∑cisMUFA**	1.21	0.97	0.99	1.06	0.10	0.48	0.11
**∑n-6 PUFA**	0.62	0.57	0.55	0.68	0.05	0.33	0.09
**∑n-3 PUFA**	0.35	0.26	0.29	0.37	0.05	0.50	0.11
**∑FA**	9.25	8.73	9.43	9.70	0.93	0.61	0.80

LNA, Linoleic acid (18:2n-6); ∑SFA, sum of saturated fatty acids; ∑cisMUFA, sum of cis-monounsaturated fatty acids; ∑n-6 PUFA, sum of omega-6 polyunsaturated fatty acids; ∑n-3 PUFA, sum of omega-3 polyunsaturated fatty acids; ∑FA, sum of total fatty acids; n.d., not detected; N/A, not applicable.

1 Data are presented as least squares means (LS means) with SEM. Linear and quadratic *P*-values represent orthogonal polynomial contrasts across increasing doses of t10-18:1.

a,b,c Within a row, LS means without a common superscript letter differ (*P* < 0.05).

**Table 3:** Effects of increasing doses of trans-10, cis-12 CLA on fatty acid composition (μg/well) of mature bovine intramuscular adipocytes.^1^

Fatty acid	0 μM	25 μM	50 μM	100 μM	SEM	Linear *P*-value	Quadratic *P*-value
**16:0**	5.16	4.88	5.32	4.68	0.23	0.22	0.36
**18:0**	3.03	2.66	3.02	2.63	0.21	0.32	0.88
**c9-16:1**	0.08	0.09	0.11	0.07	0.02	0.40	0.85
**c9-18:1**	0.73	0.70	0.68	0.64	0.06	0.78	0.68
**c10-18:1**	n.d.	n.d.	n.d.	n.d.	N/A	N/A	N/A
**t10-18:1**	n.d.	n.d.	n.d.	n.d.	N/A	N/A	N/A
**t10, c12-CLA**	0.00^a^	0.45^ab^	0.73^bc^	1.32^c^	0.17	<0.01	0.61
**LNA**	0.26	0.21	0.23	0.29	0.05	0.41	0.26
**∑SFA**	9.28	8.70	9.86	10.57	0.96	0.84	0.46
**∑cisMUFA**	1.52	1.21	1.46	1.34	0.14	0.65	0.65
**∑n-6 PUFA**	0.75	0.69	0.71	0.79	0.09	0.54	0.40
**∑n-3 PUFA**	0.42	0.44	0.52	0.45	0.05	0.56	0.17
**∑FA**	12.33	12.05	14.05	13.70	0.76	0.10	0.53

LNA, Linoleic acid (18:2n-6); ∑SFA, sum of saturated fatty acids; ∑cisMUFA, sum of cis-monounsaturated fatty acids; ∑n-6 PUFA, sum of omega-6 polyunsaturated fatty acids; ∑n-3 PUFA, sum of omega-3 polyunsaturated fatty acids; ∑FA, sum of total fatty acids; n.d., not detected; N/A, not applicable.

1 Data are presented as least squares means (LS means) with SEM. Linear and quadratic *P*-values represent orthogonal polynomial contrasts across increasing doses of trans-10, cis-12 CLA.

a,b,c Within a row, LS means without a common superscript letter differ (*P* < 0.05).

**Table 4:** Effects of increasing doses of LNA on fatty acid composition (μg/well) of mature bovine intramuscular adipocytes.^1^

Fatty acid	0 μM	25 μM	50 μM	100 μM	SEM	Linear *P*-value	Quadratic *P*-value
**16:0**	4.44	4.59	4.13	4.06	0.32	0.28	0.98
**18:0**	2.43	2.78	2.46	2.16	0.19	0.14	0.23
**c9-16:1**	0.05	0.05	0.05	0.05	0.01	0.67	0.81
**c9-18:1**	0.73	0.78	0.68	0.58	0.07	0.06	0.50
**c10-18:1**	n.d.	n.d.	n.d.	n.d.	N/A	N/A	N/A
**t10-18:1**	n.d.	n.d.	n.d.	n.d.	N/A	N/A	N/A
**t10, c12-CLA**	n.d.	n.d.	n.d.	n.d.	N/A	N/A	N/A
**LNA**	0.51^a^	1.51^ab^	3.13^bc^	4.48^c^	0.63	<0.01	0.51
**∑SFA**	7.76	8.37	7.41	7.02	0.58	0.21	0.64
**∑cisMUFA**	1.20^ab^	1.32^a^	1.12^ab^	0.93^b^	0.11	0.03	0.39
**∑n-6 PUFA**	0.97^a^	2.02^ab^	3.53^bc^	4.87^c^	0.64	<0.01	0.52
**∑n-3 PUFA**	0.42^a^	0.44^a^	0.34^ab^	0.29^b^	0.04	0.02	0.90
**∑FA**	10.57	12.38	12.67	13.33	1.13	0.12	0.47

LNA, Linoleic acid (18:2n-6); ∑SFA, sum of saturated fatty acids; ∑cisMUFA, sum of cis-monounsaturated fatty acids; ∑n-6 PUFA, sum of omega-6 polyunsaturated fatty acids; ∑n-3 PUFA, sum of omega-3 polyunsaturated fatty acids; ∑FA, sum of total fatty acids; n.d., not detected; N/A, not applicable.

1 Data are presented as least squares means (LS means) with SEM. Linear and quadratic *P*-values represent orthogonal polynomial contrasts across increasing doses of linoleic acid.

a,b,c Within a row, LS means without a common superscript letter differ (*P* < 0.05).

#### CisMUFA/SFA ratio and SCD1 indices

**Figure 3:**
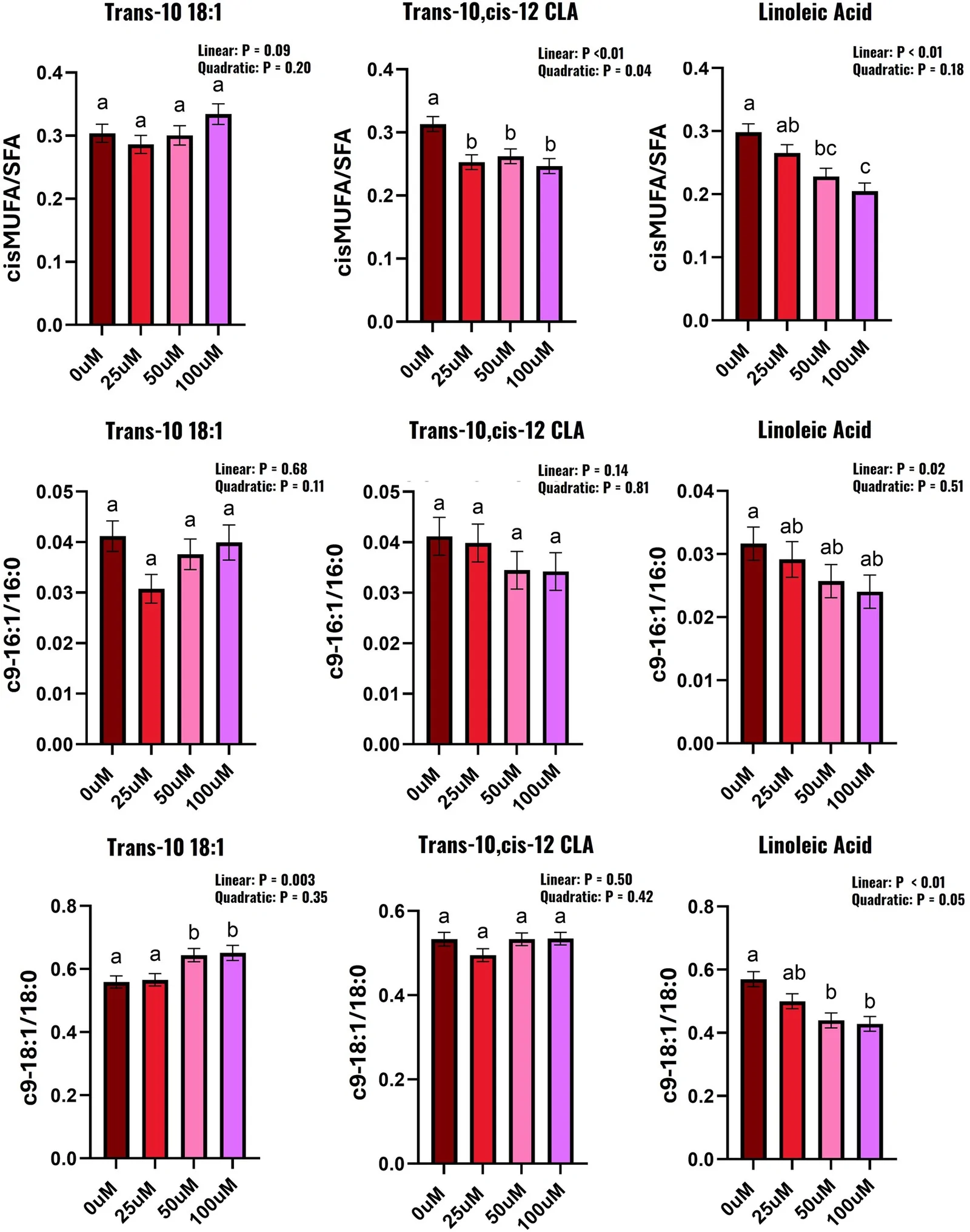
cisMUFA/SFA ratio and SCD1 indices (c9-18:1/18:0 and c9-16:1/16:0 ratios) in mature intramuscular adipocytes after treatment with increasing doses (0–100 µM) of t10-18:1, t10, c12-CLA, and linoleic acid for 9 d. Linear and quadratic dose-response *P*-values are shown above each graph. Results are shown as mean ± SEM. Bars not sharing a common letter are significantly different (*P* < 0.05).

The effects of increasing doses of the supplemental fatty acids on the cisMUFA/SFA ratio and Δ9-desaturation indices are shown in Figure 3. The cisMUFA/SFA ratio tended to increase linearly with increasing doses of t10-18:1 (*P* = 0.09). Increasing doses of t10, c12-CLA linearly decreased the cisMUFA/SFA ratio (*P *< 0.01), with a significant quadratic effect (*P* = 0.04). Compared with 0 μM (BSA control), 25, 50, and 100 μM t10, c12-CLA similarly reduced the cisMUFA/SFA ratio. Increasing doses of LNA linearly decreased the cisMUFA/SFA ratio (*P* < 0.01). To further evaluate the effects of the treatments on Δ9-desaturation, the c9-16:1/16:0 and c9-18:1/18:0 ratios were used as indirect indices of SCD1 activity. Increasing doses of t10-18:1 linearly increased the c9-18:1/18:0 ratio (*P* < 0.01) but did not affect the c9-16:1/16:0 ratio. Treatment with t10, c12-CLA did not affect either SCD1 index. Increasing doses of LNA linearly decreased both the c9-16:1/16:0 (*P* = 0.02) and c9-18:1/18:0 (*P* < 0.01) ratios (Figure 3).

#### Gene expression

**Figure 4:**
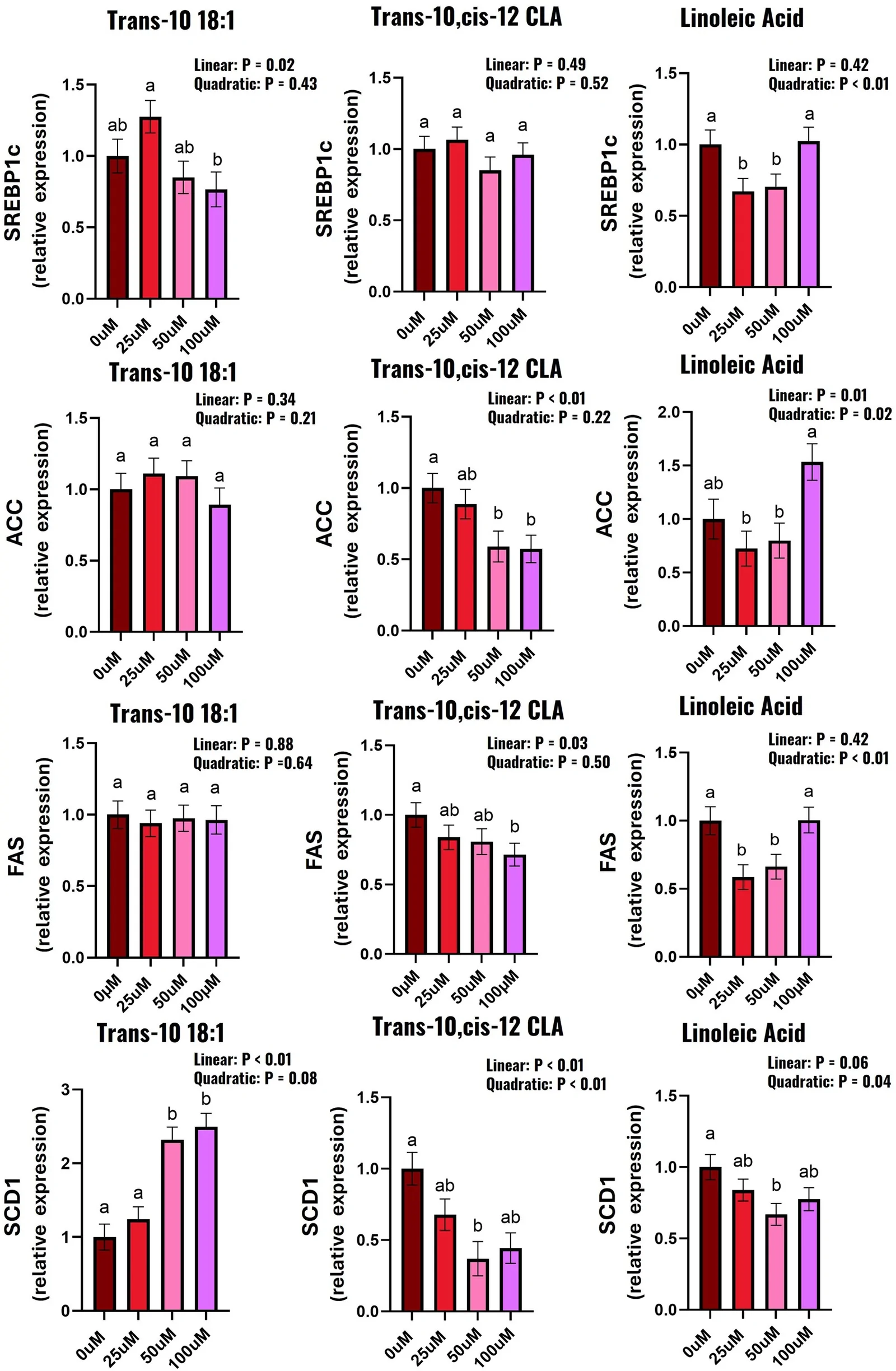
Relative mRNA expression of target genes in mature intramuscular adipocytes after 9 d of treatment with increasing doses (0–100 µM) of t10-18:1, t10, c12-CLA, and linoleic acid. Linear and quadratic dose-response *P*-values are shown above each graph. Results are shown as mean ± SEM. All data are expressed as fold change relative to the 0 µM (BSA control). Bars not sharing a common letter are significantly different (*P* < 0.05). SREBP-1c, sterol regulatory element-binding protein 1c; ACC, acetyl-CoA carboxylase; FAS, fatty acid synthase; SCD1, stearoyl-CoA desaturase..

The effects of increasing doses of the supplemental fatty acids on the mRNA expression of lipogenic genes are shown in Figure 4. SREBP1c expression exhibited a significant linear response to increasing doses of t10-18:1 (*P* = 0.02), although no individual dose differed significantly from 0 μM (BSA control). ACC and FAS expression were unaffected by t10-18:1 dose (*P* > 0.10), whereas SCD1 expression increased linearly (*P* < 0.01), with significantly greater expression at 50 and 100 μM t10-18:1 compared with 0 μM.

Increasing doses of t10, c12-CLA did not affect SREBP-1c expression but linearly decreased ACC (*P* < 0.01) and FAS (*P* = 0.03) expression. ACC expression was significantly lower at 50 and 100 μM t10, c12-CLA compared with 0 μM, whereas 25 μM did not differ from 0 μM. FAS expression was significantly lower at 100 μM t10, c12-CLA compared with 0 μM, whereas the 25 and 50 μM doses did not differ from 0 μM. SCD1 expression exhibited both linear and quadratic responses to increasing doses of t10, c12-CLA (both *P* < 0.01), with significantly lower expression at 50 μM compared with 0 μM, whereas the 25 and 100 μM doses did not differ from 0 μM (Figure 4).

Increasing doses of LNA did not linearly affect SREBP-1c or FAS expression, although both genes exhibited significant quadratic responses (both *P* < 0.01). Treatment with LNA reduced SREBP-1c expression at 25 and 50 μM compared with 0 μM, whereas the 100 μM dose did not differ from 0 μM. FAS expression was lower at 100 μM LNA than at 25 and 50 μM but did not differ from 0 μM. ACC expression exhibited both linear (*P* = 0.01) and quadratic (*P* = 0.02) responses to increasing doses of LNA. ACC expression was greater at 100 μM LNA than at 25 and 50 μM but did not differ from 0 μM (*P* > 0.10). SCD1 expression exhibited a quadratic response (*P* = 0.04) and tended to decrease linearly with increasing doses of LNA (*P* = 0.06), with only the 50 μM dose differing significantly from 0 μM (Figure 4).

**Figure 5:**
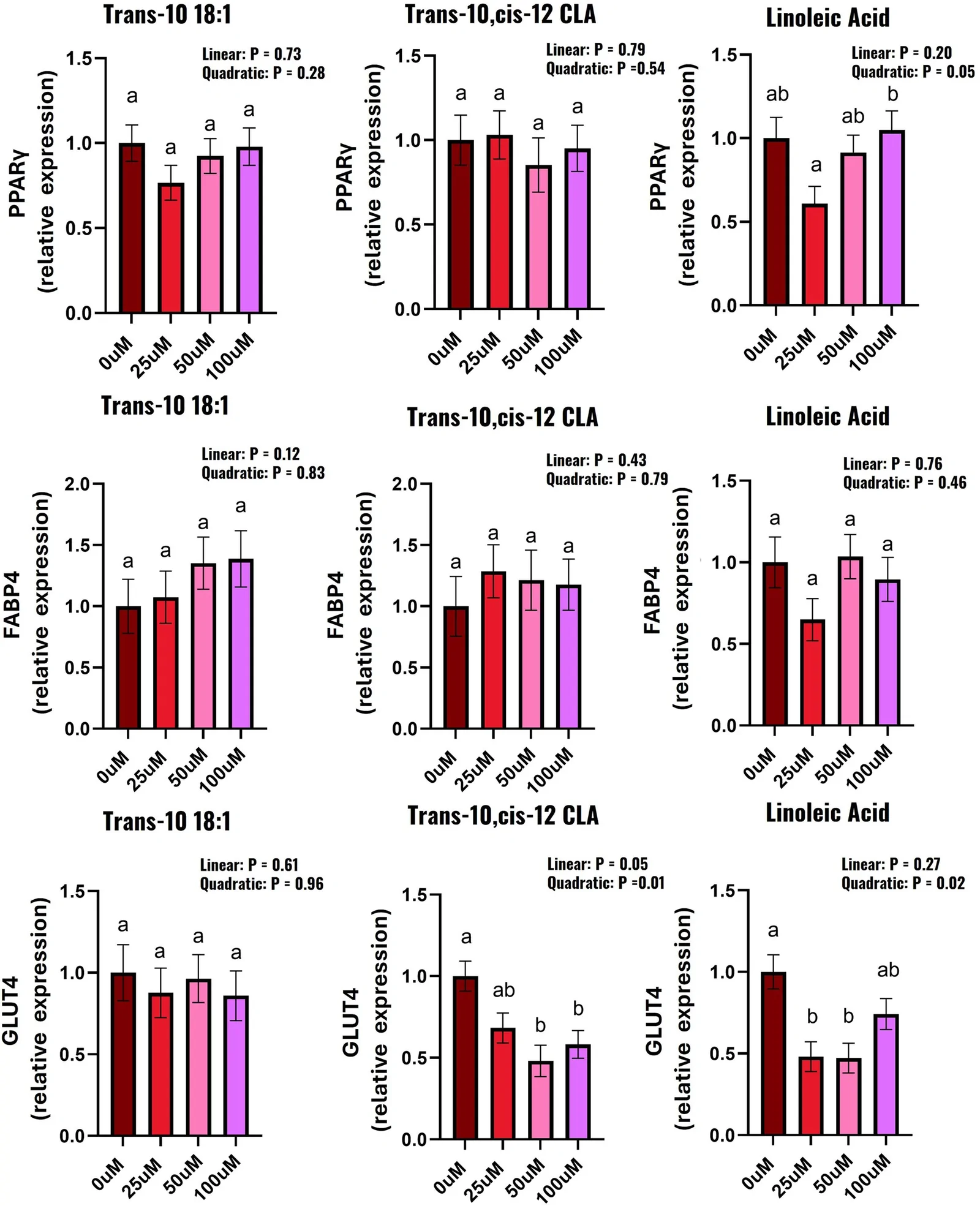
Relative mRNA expression of PPARγ, FABP4, and GLUT4 in mature intramuscular adipocytes after 9 d of treatment with increasing doses (0–100 µM) of t10-18:1, t10, c12-CLA, and linoleic acid. Linear and quadratic dose-response *P*-values are shown above each graph. Results are shown as mean ± SEM. All data are expressed as fold change relative to the 0 µM (BSA control). Bars not sharing a common letter are significantly different (*P* < 0.05). PPARγ, peroxisome proliferator-activated receptor gamma; FABP4, fatty acid-binding protein 4; GLUT4, glucose transporter type 4.

The effects of increasing doses of the supplemental fatty acids on the mRNA expression of PPARγ, FABP4, and GLUT4 are shown in Figure 5. Increasing doses of t10-18:1 did not affect PPARγ, FABP4, or GLUT4 expression (*P* > 0.10). Increasing doses of t10, c12-CLA did not affect PPARγ or FABP4 expression but resulted in both linear (*P* = 0.05) and quadratic (*P* = 0.01) decreases in GLUT4 expression. GLUT4 expression was significantly lower at 50 and 100 μM t10, c12-CLA compared with 0 μM, whereas the 25 μM dose did not differ from 0 μM.

Increasing doses of LNA did not linearly affect PPARγ expression but resulted in a quadratic response (*P* = 0.05). PPARγ expression was significantly greater at 100 μM LNA than at 25 μM but did not differ from 0 μM. FABP4 expression was unaffected by increasing doses of LNA (*P* > 0.10). GLUT4 expression did not respond linearly to increasing doses of LNA but exhibited a significant quadratic response (*P* = 0.02). GLUT4 expression was significantly lower at 25 and 50 μM LNA compared with 0 μM, whereas the 100 μM dose did not differ from 0 μM (Figure 5).

### Experiment 2: direct comparison of fatty acid treatments in differentiated adipocytes

In the second experiment, we compared the effects of LNA and its trans-10 biohydrogenation intermediates (t10, c12-CLA and t10-18:1) in differentiated adipocytes treated with 50 µM of each fatty acid for 9 d. A fourth treatment (c10-18:1) was included to determine whether the bioactivity of t10-18:1 (ie, SCD1 upregulation) is driven by double-bond geometry (cis vs trans).

#### Cellular lipid content and fatty acid composition

**Figure 6:**
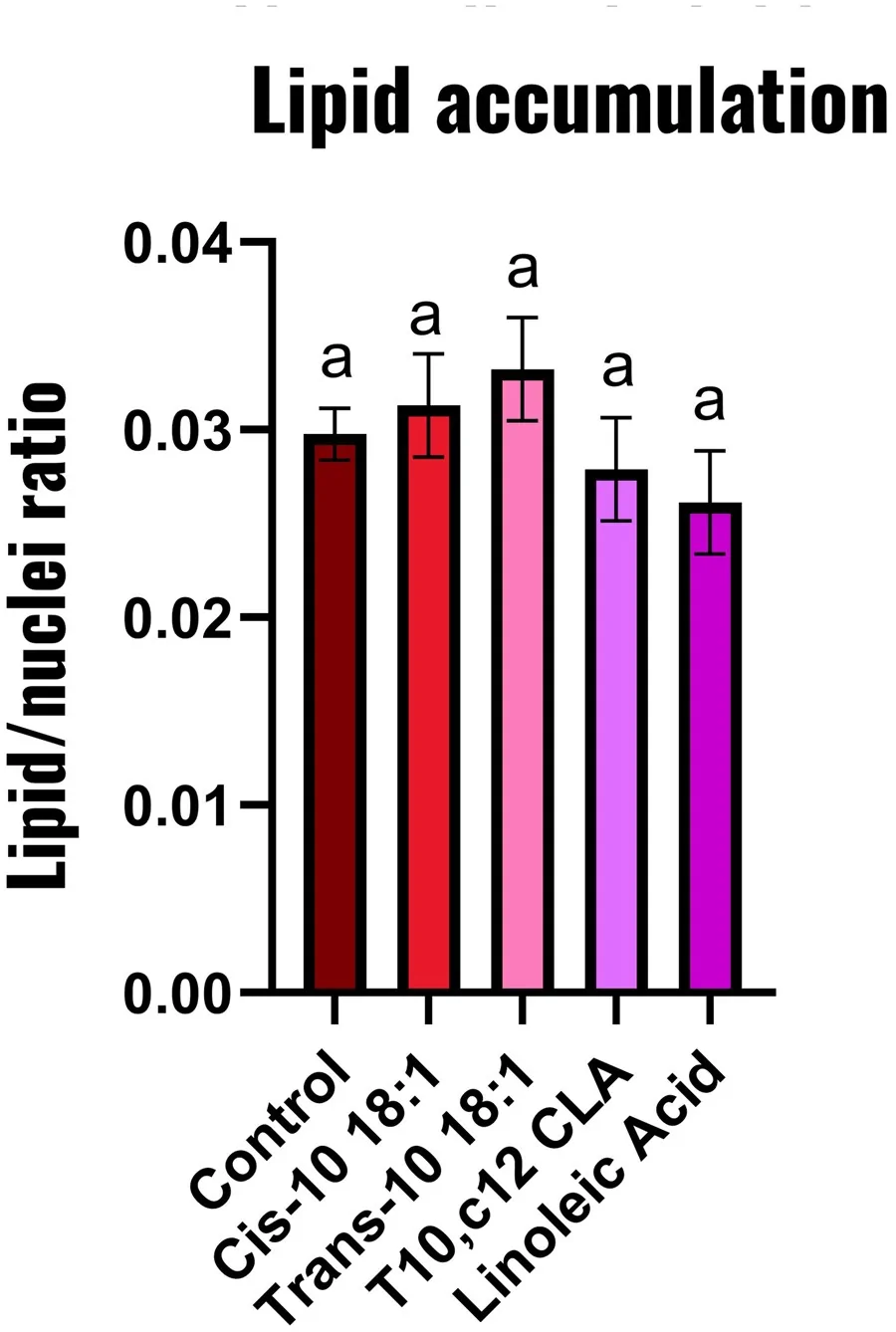
Normalized lipid accumulation (lipid/nuclei ratio) in mature intramuscular adipocytes after 9 d of treatment with BSA control or 50 µM of c10-18:1, t10-18:1, t10, c12-CLA, or linoleic acid. Results are shown as mean ± SEM. Bars sharing a common letter are not significantly different (*P* > 0.05).

After 9 d of exposure to 50 μM fatty acid treatments, normalized lipid accumulation (lipid/nuclei ratio) did not differ among treatments (Figure 6). Similarly, total fatty acid content (μg/well) was unaffected by treatment (Table 5). Treatment effects on the amounts of individual fatty acids and major fatty acid classes are also presented in Table 5. Total SFA was lower in t10-18:1-treated adipocytes than in the BSA control and t10, c12-CLA-treated adipocytes (*P* = 0.05). Treatment with t10, c12-CLA resulted in greater 18:0 (stearic acid) than t10-18:1 (*P* = 0.04), although neither treatment differed significantly from the BSA control. Treatment with c10-18:1 resulted in significantly greater total cisMUFA compared with the BSA control and all other fatty acid treatments (*P* < 0.01). Similarly, treatment with LNA resulted in significantly greater total n-6 PUFA compared with the BSA control and all other fatty acid treatments (*P* < 0.01). Total n-3 PUFA was greater in c10-18:1-treated adipocytes than in the BSA control and t10-18:1- and LNA-treated adipocytes (*P* = 0.03) but did not differ from t10, c12-CLA-treated adipocytes. No other individual fatty acids or major fatty acid classes were affected by the supplemental fatty acids (Table 5).

**Table 5:** Effects of 50 μM fatty acid treatments on fatty acid composition (μg/well) of mature bovine intramuscular adipocytes.^1^

Fatty acid	BSA control	c10-18:1	t10-18:1	t10, c12-CLA	LNA	SEM	*P*-value
**16:0**	4.32	4.50	3.28	5.32	4.07	0.53	0.18
**18:0**	2.55^ab^	2.68^ab^	1.64^b^	3.02^a^	2.44^ab^	0.30	0.04
**c9-16:1**	0.09	0.11	0.08	0.11	0.05	0.03	0.66
**c9-18:1**	0.67	0.71	0.63	0.80	0.70	0.09	0.27
**c10-18:1**	n.d.	2.15	n.d.	n.d.	n.d.	N/A	N/A
**t10-18:1**	n.d.	n.d.	1.94	n.d.	n.d.	N/A	N/A
**t10, c12-CLA**	n.d.	n.d.	n.d.	0.80	n.d.	N/A	N/A
**LNA**	0.25ᵃ	0.19ᵃ	0.22ᵃ	0.23ᵃ	3.22ᵇ	0.26	<0.01
**∑SFA**	7.90^a^	7.87^ab^	5.81^b^	9.86^a^	7.30^ab^	0.89	0.05
**∑cisMUFA**	1.32ᵃ	4.34ᵇ	1.06ᵃ	1.47ᵃ	1.13ᵃ	0.29	<0.01
**∑n-6 PUFA**	0.73^a^	0.56^a^	0.57^a^	0.71^a^	3.61^b^	0.27	<0.01
**∑n-3 PUFA**	0.37^c^	0.55^a^	0.31^c^	0.52^ab^	0.33^bc^	0.06	0.03
**∑FA**	10.70	13.52	10.05	14.05	12.62	1.21	0.08

LNA, Linoleic acid (18:2n-6); ∑SFA, sum of saturated fatty acids; ∑cisMUFA, sum of cis-monounsaturated fatty acids; ∑n-6 PUFA, sum of omega-6 polyunsaturated fatty acids; ∑n-3 PUFA, sum of omega-3 polyunsaturated fatty acids; ∑FA, sum of total fatty acids; n.d., not detected; N/A, not applicable.

1 Data are presented as least squares means (LS means) with SEM.

a,b,c Within a row, LS means without a common superscript letter differ (*P* < 0.05).

#### CisMUFA/SFA ratio and SCD1 indices

**Figure 7:**
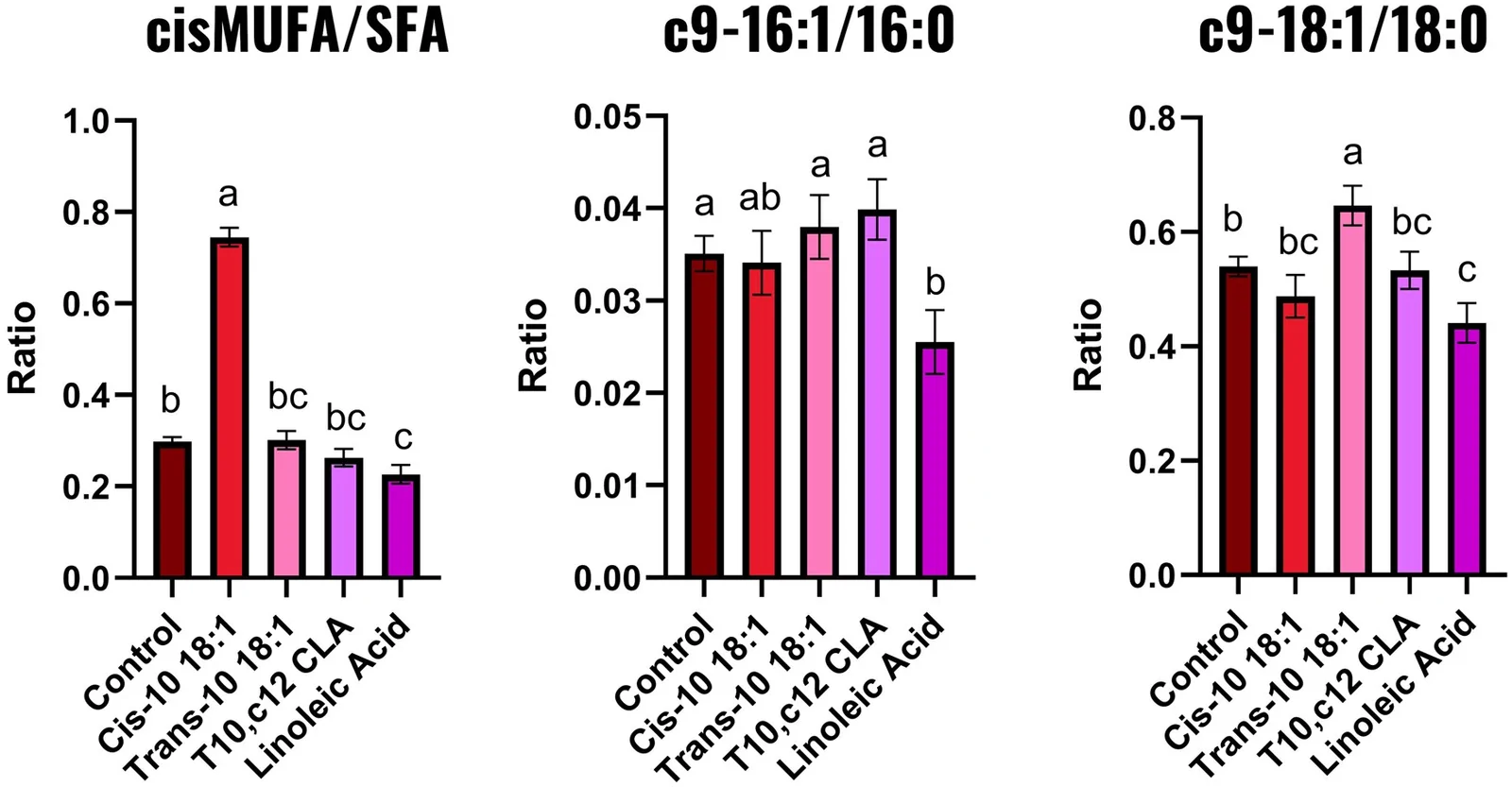
cisMUFA/SFA ratio and SCD1 indices (c9-18:1/18:0 and c9-16:1/16:0 ratios) in mature intramuscular adipocytes after treatment with BSA control or 50 µM of c10-18:1, t10-18:1, t10, c12-CLA, or LNA for 9 d. Results are shown as mean ± SEM. Bars not sharing a common letter are significantly different (*P* < 0.05).

The effects of the 50 μM supplemental fatty acid treatments on the cisMUFA/SFA ratio and Δ9-desaturation indices are shown in Figure 7. The cisMUFA/SFA ratio was greatest in c10-18:1-treated adipocytes and was significantly greater than in the BSA control and all other fatty acid treatments (*P* < 0.01), consistent with the accumulation of c10-18:1 and the resulting increase in total cisMUFA content (Table 5). LNA reduced the cisMUFA/SFA ratio compared with the BSA control (*P* < 0.05), whereas t10-18:1 and t10, c12-CLA did not differ from the BSA control. LNA reduced the c9-16:1/16:0 ratio compared with the BSA control, t10-18:1, and t10, c12-CLA treatments (*P* < 0.05) but was not different from c10-18:1 (*P* > 0.10). The c9-18:1/18:0 ratio was greater in t10-18:1-treated adipocytes than in the BSA control and all other fatty acid treatments (*P* < 0.05). LNA reduced the c9-18:1/18:0 ratio compared with the BSA control and t10-18:1 treatment (*P* < 0.05), whereas c10-18:1 and t10, c12-CLA did not differ from the BSA control (*P* > 0.10).

#### Gene expression

**Figure 8:**
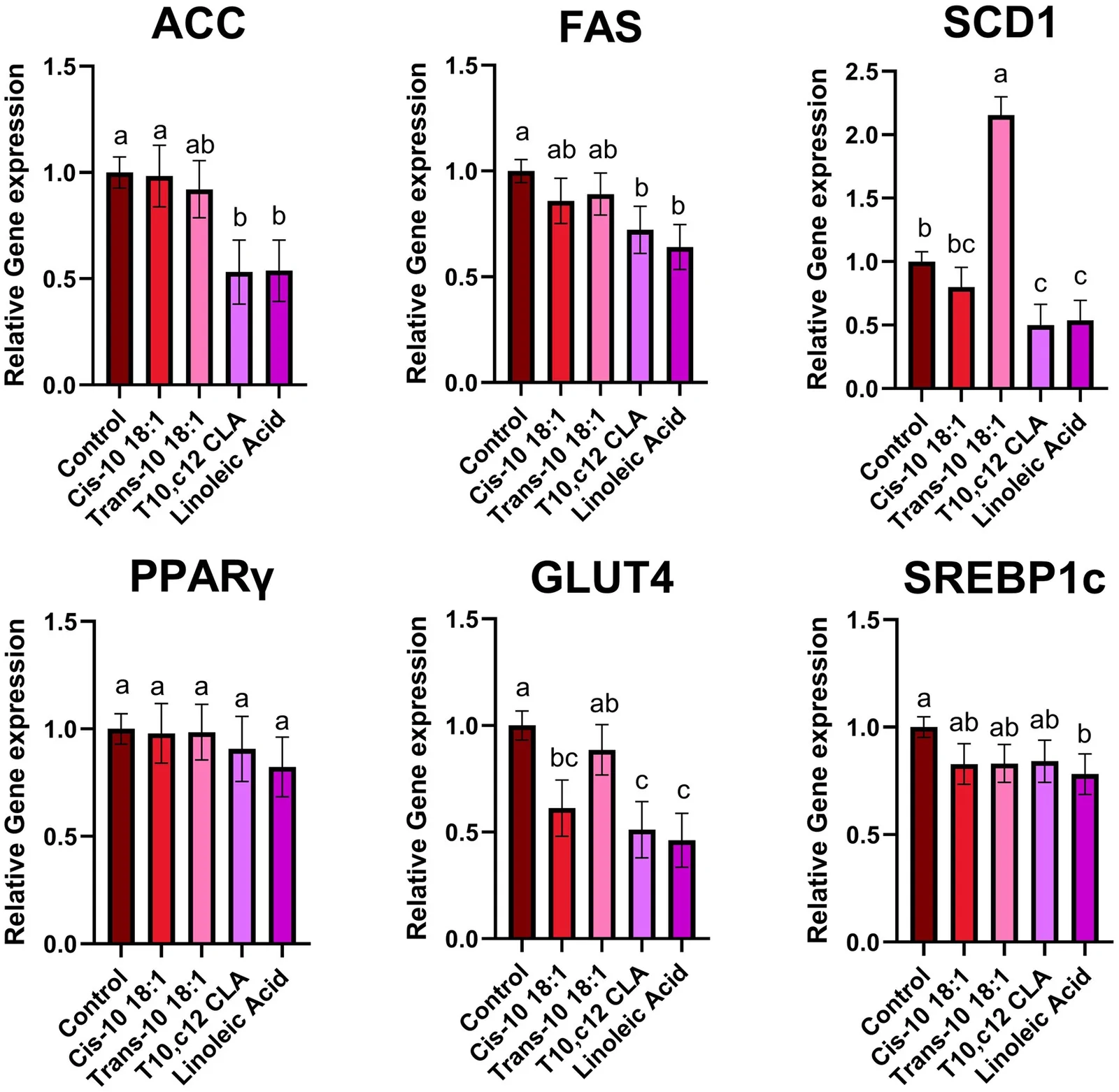
Relative mRNA expression of target genes in mature intramuscular adipocytes after 9 d of treatment with BSA control or 50 µM of c10-18:1, t10-18:1, t10, c12-CLA, or linoleic acid. Results are shown as mean ± SEM. All data are expressed as fold change relative to the 0 µM (BSA control). Bars not sharing a common letter are significantly different (*P* < 0.05). ACC, acetyl-CoA carboxylase; FAS, fatty acid synthase; SCD1, stearoyl-CoA desaturase 1; PPARγ, peroxisome proliferator-activated receptor gamma; GLUT4, glucose transporter type 4; SREBP1c, sterol regulatory element-binding protein 1c.

The effects of the 50 μM fatty acid treatments on the mRNA expression of selected genes are shown in Figure 8. Treatment effects were observed for ACC, FAS, SCD1, GLUT4, and SREBP-1c (*P* < 0.05), whereas PPARγ expression did not differ among treatments. ACC expression was lower in t10, c12-CLA- and LNA-treated adipocytes than in the BSA control and c10-18:1-treated adipocytes (*P* < 0.05), whereas t10-18:1-treated adipocytes did not differ from the BSA control or the other fatty acid treatments. Similarly, FAS expression was lower in t10, c12-CLA- and LNA-treated adipocytes than in the BSA control (*P* < 0.05), whereas c10-18:1- and t10-18:1-treated adipocytes did not differ from the BSA control or the other fatty acid treatments. SCD1 expression was ∼2-fold greater in t10-18:1-treated adipocytes than in the BSA control and 1.7- to 2.4-fold greater than with the other fatty acid treatments (*P* < 0.05). GLUT4 expression was lower in t10, c12-CLA- and LNA-treated adipocytes than in the BSA control (*P* < 0.05), whereas t10-18:1 and c10-18:1 did not differ from the BSA control or the other fatty acid treatments. LNA reduced SREBP-1c expression compared with the BSA control (*P* < 0.05), whereas the other treatments did not differ from the BSA control (Figure 8).

## Discussion

Intramuscular fat in beef develops through the proliferation (hyperplasia) of intramuscular preadipocytes, followed by their differentiation into mature adipocytes and subsequent lipid accumulation (hypertrophy) (Park et al. 2018). Hyperplasia occurs primarily from late gestation through approximately 250 d after birth, whereas adipocyte hypertrophy and lipogenesis become more prominent during the finishing stage as intramuscular fat deposition increases (Park et al. 2018; Nguyen et al. 2021). Therefore, this study focused on differentiated adipocytes to better reflect the feedlot finishing period and to evaluate the regulation of lipogenic processes by LNA and its trans-10 biohydrogenation intermediates. Experiment 1 included dose-response treatments with fatty acids (LNA, t10-18:1, and t10, c12-CLA) at concentrations up to 100 µM, a range previously used to evaluate the bioactivity of t10, c12-CLA in primary adipocyte models and shown to have no negative effects on cell viability (Kadegowda et al. 2013). For Experiment 2, a single dose of 50 µM was used to directly compare the bioactivity of LNA, t10-18:1, and t10, c12-CLA. In addition, c10-18:1 was included as a cis-isomer control for t10-18:1.

### Linoleic acid and trans-10, cis-12 CLA, but not trans-10 18:1, reduced lipogenic gene expression in primary bovine adipocytes

Both LNA and t10, c12-CLA downregulated genes involved in de novo fatty acid synthesis (FAS), Δ9-desaturation (SCD1), and glucose uptake (GLUT4) across Experiments 1 and 2. However, LNA also downregulated SREBP-1c, a master regulator of lipogenesis, in both experiments, suggesting that LNA had broader effects on lipogenic regulation than t10, c12-CLA. In contrast, t10-18:1 did not reduce the expression of any of these genes in either experiment.

The anti-lipogenic effects of t10, c12-CLA have been partly attributed to inhibition of SREBP-1c, a key regulator of lipogenic gene expression, thereby reducing the expression of downstream lipogenic genes, including ACC, FAS, and SCD1 (Obsen et al. 2012; Ladeira et al. 2018). However, t10, c12-CLA did not significantly downregulate SREBP-1c in either experiment, despite reductions in ACC, FAS, and SCD1 expression. These findings suggest that the anti-lipogenic effects of t10, c12-CLA may involve mechanisms beyond changes in SREBP-1c mRNA expression, including post-transcriptional regulation of SREBP-1c or other signaling pathways (Kennedy et al. 2010).

In contrast, LNA reduced the expression of SREBP-1c, FAS, SCD1, and GLUT4 in both experiments 1 and 2. Polyunsaturated fatty acids, including LNA, are known to suppress lipogenic gene transcription, including ACC, FAS, and SCD1, through downregulation of sterol regulatory element-binding proteins (Worgall et al. 1998; Xu et al. 1999; Yahagi et al. 1999; Madsen et al. 2005; Teran-Garcia et al. 2007). Given the consistent downregulation of SREBP-1c and its target genes by LNA in the present study, it is plausible that the anti-lipogenic effects of LNA at the gene expression level are mediated, at least in part, via SREBP-1c-dependent mechanisms.

Glucose is a major substrate for de novo lipogenesis in intramuscular adipose tissue, and GLUT4 is a key insulin-responsive glucose transporter involved in glucose utilization during intramuscular fat development in beef cattle (Ladeira et al. 2018). In the present study, GLUT4 expression was downregulated with both LNA and t10, c12-CLA. Consistent with these findings, LNA and t10, c12-CLA have been shown to suppress GLUT4 expression human adipocytes and skeletal muscle cells, respectively (Brown and McIntosh 2003; Kennedy et al. 2008; Poletto et al. 2015). The reduced GLUT4 expression by t10, c12-CLA has been attributed to its antagonism of PPARγ signaling, whereby t10, c12-CLA may indirectly reduce GLUT4 expression and insulin responsiveness (Brown and McIntosh 2003; Kennedy et al. 2008). LNA, on the other hand, has been shown to downregulate GLUT4 expression via reduced SREBP1 transcriptional activity (Im et al. 2006; Poletto et al. 2015). However, gene expression changes alone cannot confirm altered substrate utilization, and direct tracer-based studies using labeled glucose should be explored to verify effects on glucose utilization and de novo lipogenesis in bovine adipocytes.

### Unchanged lipid accumulation despite reduced lipogenic gene expression

Despite reduced expression of lipogenic genes following LNA and t10, c12-CLA treatment, total lipid accumulation in adipocytes remained unchanged. One explanation may relate to the timing of fatty acid treatment relative to adipogenesis and lipogenesis. Most studies reporting inhibitory effects of t10, c12-CLA in adipocytes applied treatments during the differentiation phase, when t10, c12-CLA can inhibit adipogenesis through key adipogenic regulators such as PPARγ and CCAAT/enhancer-binding proteins (C/EBPs) (Kennedy et al. 2010). In contrast, in the present study, treatments were applied post-differentiation to specifically assess effects on lipogenesis in mature adipocytes. Consequently, potential inhibitory effects of fatty acid treatment on adipogenesis during differentiation, and the resulting downstream effects on lipid accumulation, were not captured in the present study.

An additional explanation for the lack of change in lipid accumulation may be the compensatory accumulation of exogenous fatty acids from the culture medium. Fatty acid analysis confirmed dose-dependent accumulation of supplemented fatty acids in cellular lipids across all treatments, including t10-18:1 (Table 2), LNA (Table 3), and t10, c12-CLA (Table 4). Similar dose-dependent accumulation of exogenous fatty acids in adipocytes has been reported previously (Otto and Lane 2005; Małodobra-Mazur et al. 2024). Therefore, the reduction in de novo lipogenesis induced by LNA and t10, c12-CLA may have been offset by the accumulation of supplemented fatty acids in cellular lipids, thereby maintaining overall lipid accumulation.

### Linoleic acid is a more potent inhibitor of Δ9-desaturation than trans-10, cis-12 CLA in primary bovine intramuscular adipocytes

One consistent effect of t10, c12-CLA in both in vivo and adipocyte culture studies is a reduction in the cisMUFA/SFA ratio, which has been attributed to the inhibitory effect of t10, c12-CLA on SCD1, a key rate-limiting enzyme that catalyzes the Δ9 desaturation of SFA to cisMUFA (Ntambi 1999; Gillis et al. 2004; Schlegel et al. 2012; Kadegowda et al. 2013; Liu et al. 2018; Vahmani et al. 2025). In the present study, both LNA and t10, c12-CLA downregulated SCD1 expression. However, only LNA consistently reduced the cisMUFA/SFA ratio and both the c9-18:1/18:0 and c9-16:1/16:0 Δ9-desaturation indices, indicating a greater inhibitory effect on Δ9-desaturation than t10, c12-CLA. Similar effects of LNA have been reported in non-ruminant adipocyte studies, where it has been attributed to reduced SCD1 mRNA abundance and SREBP-mediated inhibition of SCD1 gene expression (Ntambi 1999; Obsen et al. 2012). Consistent with this, we observed downregulation of SREBP-1c in adipocytes treated with 25 and 50 µM of LNA.

The inhibitory effects of LNA on SCD1 expression, together with reductions in SCD1 indices and the cisMUFA/SFA ratio in adipocytes, suggest that LNA may play an equal or even more important role than t10, c12-CLA in regulating SCD1 expression and activity. Given the substantially higher circulating and tissue concentrations of LNA relative to t10, c12-CLA, it is plausible that LNA is a major contributor to the regulation of the cisMUFA/SFA ratio in intramuscular fat of feedlot cattle, particularly in animals fed LNA-rich diets. However, these findings require validation in in vivo studies.

### Trans-10 18:1 upregulates Δ9-desaturation in primary bovine intramuscular adipocytes

A notable finding of this study was the strong upregulation of SCD1 by t10-18:1 in both Experiment 1 (dose–response) and Experiment 2 (50 µM), which was accompanied by an increased c9-18:1/18:0 ratio. The comparison with c10-18:1 further demonstrated that trans geometry is critical for bioactivity, as only t10-18:1, but not the cis isomer, increased SCD1 expression and the c9-18:1/18:0 ratio. Previous studies have similarly reported that t9- and t10-18:1 isomers can increase SCD1 expression and Δ9-desaturation indices in human aortic smooth muscle cells and HepG2 hepatoma cells (Minville-Walz et al. 2012; Vahmani et al. 2016a). One explanation is these t-18:1 isomers (e.g., t9- and t10-18:1), which cannot undergo Δ9-desaturation due to the position of the double bond, may induce a compensatory upregulation of SCD1 to maintain membrane fluidity, given the higher melting point of trans fatty acids compared with cisMUFA (Ntambi 1999).

## Conclusions

Our results demonstrate that LNA and its trans-10 biohydrogenation intermediates exert distinct effects on lipogenic gene expression and the cisMUFA/SFA ratio in differentiated primary intramuscular adipocytes. Both LNA and t10, c12-CLA downregulated the expression of lipogenic genes involved in de novo fatty acid synthesis (FAS), Δ9-desaturation (SCD1), and glucose uptake (GLUT4). However, only LNA consistently reduced the cisMUFA/SFA ratio and Δ9-desaturation indices, indicating greater inhibition of Δ9-desaturation. In contrast, t10-18:1 had minimal effects on most lipogenic genes but significantly increased SCD1 expression and the c9-18:1/18:0 ratio. These findings suggest that LNA may be a more potent inhibitor of Δ9-desaturation than t10, c12-CLA in primary bovine intramuscular adipocytes under the in vitro conditions of this study. In addition, our findings raise the possibility that t10-18:1 may, to some extent, counteract the inhibitory effects of LNA and t10, c12-CLA on Δ9-desaturation by upregulating SCD1 expression. Future studies should investigate combination treatments and validate these findings in vivo to determine their relevance to intramuscular fat composition in cattle.

## Abbreviations

cisMUFA: sum of cis-monounsaturated fatty acids
∑FA: sum of total fatty acids
∑n-3 PUFA: sum of omega-3 polyunsaturated fatty acids
∑n-6 PUFA: sum of omega-6 polyunsaturated fatty acids
∑SFA: sum of saturated fatty acids
ACC: acetyl-CoA carboxylase
BSA: bovine serum albumin
cis-MUFA: cis-monounsaturated fatty acid
CLA: conjugated linoleic acid
FABP4: fatty acid-binding protein 4
FAME: fatty acid methyl ester
FAS: fatty acid synthase
GAPDH: glyceraldehyde-3-phosphate dehydrogenase
GLUT4: glucose transporter type 4
IBMX: 3-isobutyl-1-methylxanthine
LNA: linoleic acid
MUFA: monounsaturated fatty acid
PPARγ: peroxisome proliferator-activated receptor gamma
PUFA: polyunsaturated fatty acid
SCD1: stearoyl-CoA desaturase 1
SFA: saturated fatty acid
SREBP-1c: sterol regulatory element-binding protein 1c
UXT: ubiquitously expressed prefoldin-like chaperone

## References

[skag268-B1] Alves SP , Santos-Silva J, Cabrita ARJ, Fonseca AJM, Bessa RJB. 2013. Detailed dimethylacetal and fatty acid composition of rumen content from lambs fed lucerne or concentrate supplemented with soybean oil. PLoS One. 8:e58386. 10.1371/journal.pone.0058386 23484024 PMC3587585

[skag268-B2] Alves SP et al 2021. Trans-10 18:1 in ruminant meats: a review. Lipids. 56:539–562. 10.1002/lipd.12324 34608647

[skag268-B3] Brown JM , McIntosh MK. 2003. Conjugated linoleic acid in humans: regulation of adiposity and insulin sensitivity. J Nutr. 133:3041–3046. 10.1093/jn/133.10.3041 14519781 PMC1307498

[skag268-B4] Choi SH et al 2016. The expression of adipogenic genes in adipose tissues of feedlot steers fed supplementary palm oil or soybean oil. Asian-Australas J Anim Sci. 29:404–412. 10.5713/ajas.15.0011 26950873 PMC4811793

[skag268-B5] Chung KY , Choi CB, Kawachi H, Yano H, Smith SB. 2006. Trans-10, cis-12 conjugated linoleic acid antagonizes arginine-promoted differentiation of bovine preadipocytes. Adipocytes. 2:93–100. https://www.researchgate.net/publication/286050477_Trans-10_cis-12_conjugated_linoleic_acid_antagonizes_arginine-promoted_differentiation_of_bovine_preadipocytes

[skag268-B6] Evans M et al 2001. Trans-10, cis-12 conjugated linoleic acid reduces triglyceride content while differentially affecting peroxisome proliferator activated receptor γ2 and aP2 expression in 3T3-L1 preadipocytes. Lipids. 36:1223–1232. 10.1007/s11745-001-0836-z 11795855

[skag268-B7] Gillis MH , Duckett SK, Sackmann JR. 2004. Effects of supplemental rumen-protected conjugated linoleic acid or corn oil on fatty acid composition of adipose tissues in beef cattle. J Anim Sci. 82:1419–1427. 10.2527/2004.8251419x 15144082

[skag268-B8] Gotoh T , Joo ST. 2016. Characteristics and health benefit of highly marbled Wagyu and Hanwoo beef. Korean J Food Sci Anim Resour. 36:709–718. 10.5851/kosfa.2016.36.6.709 28115881 PMC5243954

[skag268-B9] Im SS et al 2006. Regulation of GLUT4 gene expression by SREBP-1c in adipocytes. Biochem J. 399:131–139. 10.1042/BJ20060696 16787385 PMC1570175

[skag268-B10] Joseph SJ et al 2010. Effect of diet supplementation on the expression of bovine genes associated with fatty acid synthesis and metabolism. Bioinform Biol Insights. 4:19–31. 10.4137/BBI.S4168 20448844 PMC2865165

[skag268-B11] Jurek S et al 2020. Optimizing adipogenic transdifferentiation of bovine mesenchymal stem cells: a prominent role of ascorbic acid in FABP4 induction. Adipocyte. 9:35–50. 10.1080/21623945.2020.1720480 31996081 PMC6999845

[skag268-B12] Kadegowda AKG , Burns TA, Pratt SL, Duckett SK. 2013. Inhibition of stearoyl-CoA desaturase 1 reduces lipogenesis in primary bovine adipocytes. Lipids. 48:967–976. 10.1007/s11745-013-3823-1 23929455

[skag268-B13] Kennedy A , Chung S, LaPoint K, Fabiyi O, McIntosh MK. 2008. Trans-10, cis-12 conjugated linoleic acid antagonizes ligand-dependent PPARγ activity in primary cultures of human adipocytes. J Nutr. 138:455–461. 10.1093/jn/138.3.455 18287349 PMC2366092

[skag268-B14] Kennedy A et al 2010. Antiobesity mechanisms of action of conjugated linoleic acid. J Nutr Biochem. 21:171–179. 10.1016/j.jnutbio.2009.08.003 19954947 PMC2826589

[skag268-B15] Kramer JKG , Hernandez M, Cruz-Hernandez C, Kraft J, Dugan MER. 2008. Combining results of two GC separations partly achieves determination of all cis and trans 16:1, 18:1, 18:2 and 18:3 except CLA isomers of milk fat as demonstrated using Ag-ion SPE fractionation. Lipids. 43:259–273. 10.1007/s11745-007-3143-4 18214567

[skag268-B16] Ladeira MM et al 2018. Review: Nutrigenomics of marbling and fatty acid profile in ruminant meat. Animal. 12: S282–S294. 10.1017/S1751731118001933 30139403

[skag268-B17] Lehnen TE , da Silva MR, Camacho A, Marcadenti A, Lehnen AM. 2015. A review on effects of conjugated linoleic fatty acid (CLA) upon body composition and energetic metabolism. J Int Soc Sports Nutr. 12:36. 10.1186/s12970-015-0097-4 26388708 PMC4574006

[skag268-B18] Liu H et al 2018. Effect of dietary conjugated linoleic acid supplementation during late gestation on colostrum yield, fatty acid composition, and IgG concentrations in primiparous sows. Can J Anim Sci. 98:732–740. 10.1139/cjas-2017-0202

[skag268-B19] Madsen L , Petersen RK, Kristiansen K. 2005. Regulation of adipocyte differentiation and function by polyunsaturated fatty acids. Biochim Biophys Acta. 1740:266–286. 10.1016/j.bbadis.2005.03.001 15949694

[skag268-B20] Małodobra-Mazur M , Ołdakowska M, Dobosz T. 2024. Exploring PPAR gamma and PPAR alpha’s regulation role in metabolism via epigenetics mechanism. Biomolecules. 14:1445. 10.3390/biom14111445 39595621 PMC11591816

[skag268-B21] Minville-Walz M et al 2012. Distinct regulation of stearoyl-CoA desaturase 1 gene expression by cis and trans C18:1 fatty acids in human aortic smooth muscle cells. Genes Nutr. 7:209–216. 10.1007/s12263-011-0258-2 22057664 PMC3316751

[skag268-B22] Nguyen DV , Nguyen OC, Malau-Aduli AEO. 2021. Main regulatory factors of marbling level in beef cattle. Vet Anim Sci. 14:100219. 10.1016/j.vas.2021.100219 34877434 PMC8633366

[skag268-B23] Ntambi JM. 1999. Regulation of stearoyl-CoA desaturase by polyunsaturated fatty acids and cholesterol. J Lipid Res. 40:1549–1558. 10.1016/S0022-2275(20)33401-5 10484602

[skag268-B24] Obsen T et al 2012. Trans-10, cis-12 conjugated linoleic acid decreases de novo lipid synthesis in human adipocytes. J Nutr Biochem. 23:580–590. 10.1016/j.jnutbio.2011.02.014 21775116 PMC3203310

[skag268-B25] Oliveira DM et al 2014. Expression of genes involved in lipid metabolism in the muscle of beef cattle fed soybean or rumen-protected fat, with or without monensin supplementation. J Anim Sci. 92:5426–5436. 10.2527/jas.2014-7855 25403202

[skag268-B26] Otto TC , Lane MD. 2005. Adipose development: from stem cell to adipocyte. Crit Rev Biochem Mol Biol. 40:229–242. 10.1080/10409230591008189 16126487

[skag268-B27] Park SJ et al 2018. Genetic, management, and nutritional factors affecting intramuscular fat deposition in beef cattle—a review. Asian-Australas J Anim Sci. 31:1043–1061. 10.5713/ajas.18.0310 29879830 PMC6039335

[skag268-B28] Poletto AC et al 2015. Oleic and linoleic fatty acids downregulate Slc2a4/GLUT4 expression via NFκB and SREBP1 in skeletal muscle cells. Mol Cell Endocrinol. 401:65–72. 10.1016/j.mce.2014.12.001 25486510

[skag268-B29] Schlegel G et al 2012. Influence of a rumen-protected conjugated linoleic acid mixture on carcass traits and meat quality in young Simmental heifers. J Anim Sci. 90:1532–1540. 10.2527/jas.2011-3617 22573839

[skag268-B30] Schmittgen TD , Livak KJ. 2008. Analyzing real-time PCR data by the comparative CT method. Nat Protoc. 3:1101–1108. 10.1038/nprot.2008.73 18546601

[skag268-B31] Smith SB. 2024. Oleic acid concentration in bovine adipose tissues: impact on human health, sensory attributes, and genetic regulation. Front Anim Sci. 5:1332861. 10.3389/fanim.2024.1332861

[skag268-B32] Smith SB , Kawachi H, Choi CB, Choi CW, Wu G, Sawyer JE. 2009. Cellular regulation of bovine intramuscular adipose tissue development and composition. J Anim Sci. 87: E72–E82. 10.2527/jas.2008-1340 18997081

[skag268-B33] Smith SB et al 2006. Adiposity, fatty acid composition, and delta-9 desaturase activity during growth in beef cattle. Anim Sci J. 77:478–486. 10.1111/j.1740-0929.2006.00375.x

[skag268-B34] Smith SB , Lunt DK, Smith DR, Walzem RL. 2020. Producing high-oleic acid beef and the impact of ground beef consumption on risk factors for cardiovascular disease: a review. Meat Sci. 163:108076. 10.1016/j.meatsci.2020.108076 32066000

[skag268-B35] Teixeira PD et al 2017. Subspecies and diet affect the expression of genes involved in lipid metabolism and chemical composition of muscle in beef cattle. Meat Sci. 133:110–118. 10.1016/j.meatsci.2017.06.009 28666109

[skag268-B36] Teran-Garcia M et al 2007. Polyunsaturated fatty acid suppression of fatty acid synthase (FASN): evidence for dietary modulation of NF-Y binding to the FASN promoter by SREBP-1c. Biochem J. 402:591–600. 10.1042/BJ20061722 17313375 PMC1863568

[skag268-B37] Vahmani P et al 2021. Changes in the fatty acid composition of steer subcutaneous fat, including biohydrogenation products, are minimal when finished on combinations of corn and barley grains and silages. Can J Anim Sci. 101:362–369. 10.1139/cjas-2020-0013

[skag268-B38] Vahmani P et al 2016a. A trans-10 18:1 enriched fraction from beef fed a barley grain-based diet induces lipogenic gene expression and reduces viability of HepG2 cells. Biochem Biophys Rep. 7:84–90. 10.1016/j.bbrep.2016.05.018 28955893 PMC5613299

[skag268-B39] Vahmani P , Rolland DC, Gzyl KE, Dugan MER. 2016b. Non-conjugated cis/trans 18:2 in beef fat are mainly Δ9-desaturation products of trans-18:1 isomers. Lipids. 51:1427–1433. 10.1007/s11745-016-4207-0 27853932

[skag268-B40] Vahmani P et al 2017. Effects of feeding steers extruded flaxseed on its own before hay or mixed with hay on animal performance, carcass quality, and meat and hamburger fatty acid composition. Meat Sci. 131:9–17. 10.1016/j.meatsci.2017.04.008 28448838

[skag268-B41] Vahmani P , Xu Y, Dugan MER, Hackmann TJ. 2025. Trans-10 shifted ruminal biohydrogenation and its implications for ruminant milk and meat fat content and quality. Can J Anim Sci. 105:1–10. 10.1139/cjas-2024-0028

[skag268-B42] Worgall TS , Sturley SL, Seo T, Osborne TF, Deckelbaum RJ. 1998. Polyunsaturated fatty acids decrease expression of promoters with sterol regulatory elements by decreasing levels of mature sterol regulatory element-binding protein. J Biol Chem. 273:25537–25540. 10.1074/jbc.273.40.25537 9748213

[skag268-B43] Xu J , Nakamura MT, Cho HP, Clarke SD. 1999. Sterol regulatory element binding protein-1 expression is suppressed by dietary polyunsaturated fatty acids: a mechanism for the coordinate suppression of lipogenic genes by polyunsaturated fats. J Biol Chem. 274:23577–23583. 10.1074/jbc.274.33.23577 10438539

[skag268-B44] Yahagi N et al 1999. A crucial role of sterol regulatory element-binding protein-1 in the regulation of lipogenic gene expression by polyunsaturated fatty acids. J Biol Chem. 274:35840–35844. 10.1074/jbc.274.50.35840 10585468

[skag268-B45] Yanting C et al 2018. Dose- and type-dependent effects of long-chain fatty acids on adipogenesis and lipogenesis of bovine adipocytes. J Dairy Sci. 101:1601–1615. 10.3168/jds.2017-13312 29153512

